# The clinical triad: a structured approach to diagnosing peripheral nerve compressions

**DOI:** 10.1007/s00264-025-06452-0

**Published:** 2025-03-07

**Authors:** Elisabet Hagert

**Affiliations:** 1 Aspetar Orthopedic and Sports Medicine Hospital, Doha, Qatar; 2 School of Medicine, Qatar University, Doha, Qatar; 3 Dept of Clinical Science and Education, Karolinska Institutet, Stockholm, Sweden

**Keywords:** Carpal tunnel syndrome, Nerve compression syndromes, Peripheral Nerve, Manual muscle testing, Triad

## Abstract

****Purpose**:**

Peripheral nerve compression syndromes are a common cause of pain, weakness, and functional limitations, yet they often remain underdiagnosed due to the limitations of traditional diagnostic methods such as electromyography and imaging. This article describes the clinical triad—manual muscle testing (MMT), sensory-collapse testing (SCT), and pain evaluation—as a structured, integrative approach to improving the diagnosis of nerve compressions.

****Methods**:**

This narrative review examines the anatomical basis and diagnostic application of the clinical triad across common peripheral nerve compression syndromes. The review focuses on the median, ulnar, and radial nerves in the upper extremity, as well as the peroneal nerve in the lower extremity. Each component of the triad is analyzed for its role in detecting nerve compressions, including the reliability of MMT for identifying muscle weakness patterns, the specificity of SCT as a confirmatory tool, and the role of pain assessment in localizing entrapment sites.

****Results**:**

The clinical triad provides a structured and accessible diagnostic framework that enhances the detection of nerve compressions, even in early-stage presentations that may evade standard diagnostic tools. It demonstrates adaptability to complex cases, including double- and multiple-crush syndromes, and offers a non-invasive, cost-effective alternative to traditional diagnostic approaches.

****Conclusion**:**

The clinical triad enhances diagnostic precision in peripheral nerve compression syndromes by integrating motor, sensory, and pain assessments. Its structured methodology facilitates early detection and targeted interventions, potentially improving patient outcomes while reducing reliance on invasive or resource-intensive diagnostic methods.

**Supplementary Information:**

The online version contains supplementary material available at 10.1007/s00264-025-06452-0.

## Background

Nerve compression syndromes, characterized by the mechanical and often dynamic compression of peripheral nerves, are a significant yet frequently under appreciated cause of pain, muscle weakness, and functional impairment. These conditions often disrupt daily activities and diminish quality of life. Traditional diagnostic methods, including electromyography (EMG) and imaging techniques, have limitations, particularly in detecting nerve compressions in their early stages.

Electrodiagnostic studies (EDX) are commonly employed to evaluate suspected nerve entrapments, particularly in the upper extremities. However, their sensitivity and specificity, especially for conditions beyond carpal and cubital tunnel syndromes, are limited, typically ranging between 30 and 65% [1, 2]. EDX may also struggle to detect mixed nerve injuries and is unable to provide comprehensive muscle function assessments or identify early-stage compressions. Therefore, clinical examination techniques should be integrated with or prioritized over sole reliance on EDX.

This article focuses on the pivotal role of clinical examination techniques, emphasizing the clinical triad of muscle, sensory, and pain testing in diagnosing nerve compression syndromes.

## The clinical triad of nerve compressions

In the early stages of nerve compression, dynamic pressure on the nerve can disrupt saltatory conduction and lead to dynamic nerve ischaemia. Mackinnon and colleagues coined the term "*Sunderland Zero*" to describe this level of nerve impairment, characterized by dynamic nerve compression without axonal loss [3]. These compressions typically affect superficial nerve fascicles at the site of pressure, resulting in sensory or motor deficits—or both—depending on the nerve segment affected.

To diagnose nerve compressions effectively, clinical evaluations must assess both motor and sensory function while identifying pain at the compression site. A clinical triad for a structured assessment of median nerve compressions was first described in 2013 [4], and has since been described in upper extremity nerve compression syndromes [5]. The triad includes:

- **Manual muscle testing**: From proximal to distal, to identify the level of compression.
- **Sensory provocative testing**: Using methods such as the sensory-collapse test (SCT) to confirm compression levels.
- **Pain testing**: Evaluating for allodynia at the site of compression to further verify the diagnosis.

## Muscle testing algorithm

The principles of manual muscle testing (MMT) were first described by Sir Herbert Seddon in 1954 [6]. MMT enables clinicians to identify specific patterns of weakness, aiding in localizing the level of nerve compression. Muscle strength is graded on a scale from M0 (no muscle power) to M5 (full strength against maximum resistance), with particular attention to the M4 grade:

- **M4-**: Power against slight resistance.
- **M4**: Power against moderate resistance.
- **M4 + **: Strong power but not at maximum resistance.

Clinicians must actively evaluate for M4 weaknesses, which can provide critical diagnostic information about nerve compressions.

## Upper extremity muscle testing algorithm

**Fig. 1:**
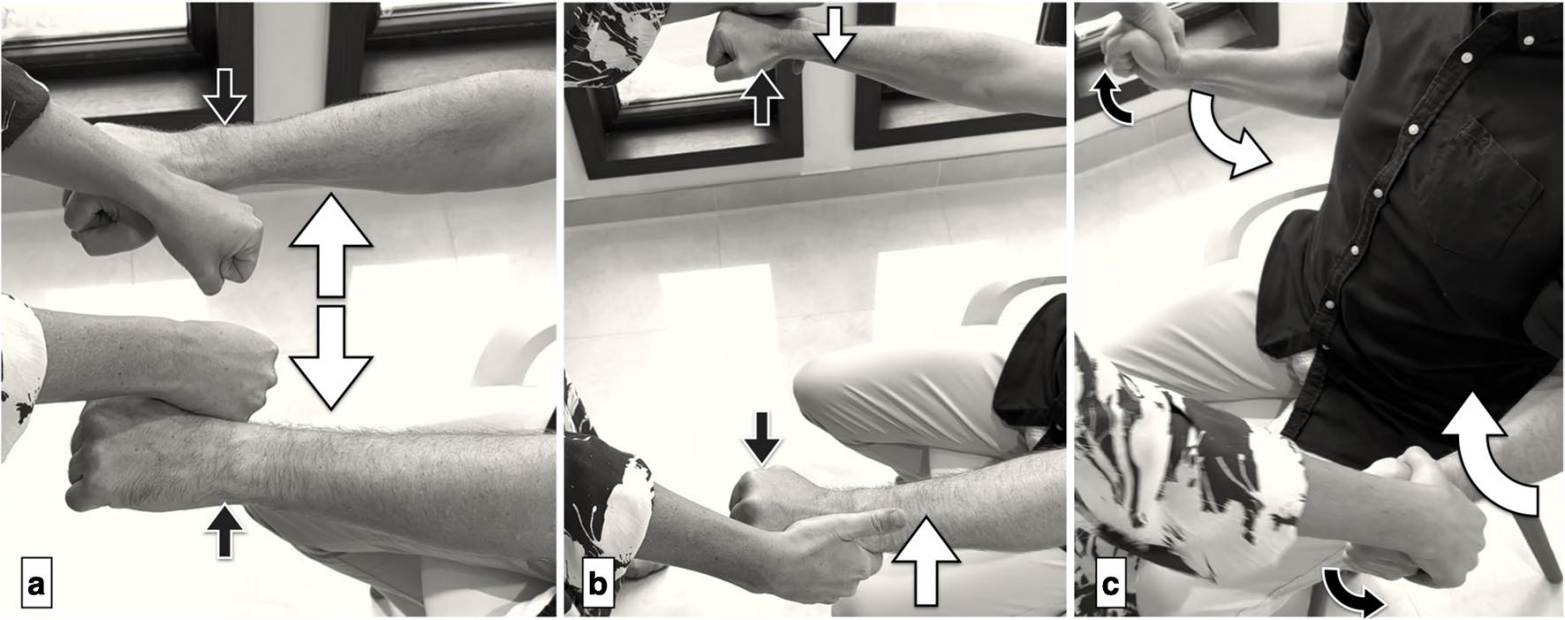
Muscle evaluation in shoulder positions. a) Testing shoulder adduction (pectorals) involves the patient keeping their arms extended while the examiner applies outward force (white arrows) and the patient resists by pressing inward (black arrows). b) Shoulder abduction (posterior deltoid) is assessed with arms straight and shoulders abducted. The examiner pushes against the outer edges of the patient’s hands (white arrows), and the patient resists by pushing outward (black arrows). c) External shoulder rotation is tested with arms positioned close to the body and elbows bent at 90 degrees. The patient attempts to rotate outward (black arrows), while the examiner applies inward pressure (white arrows). *Reprinted with permission from Aspetar Journal*

**Fig. 2:**
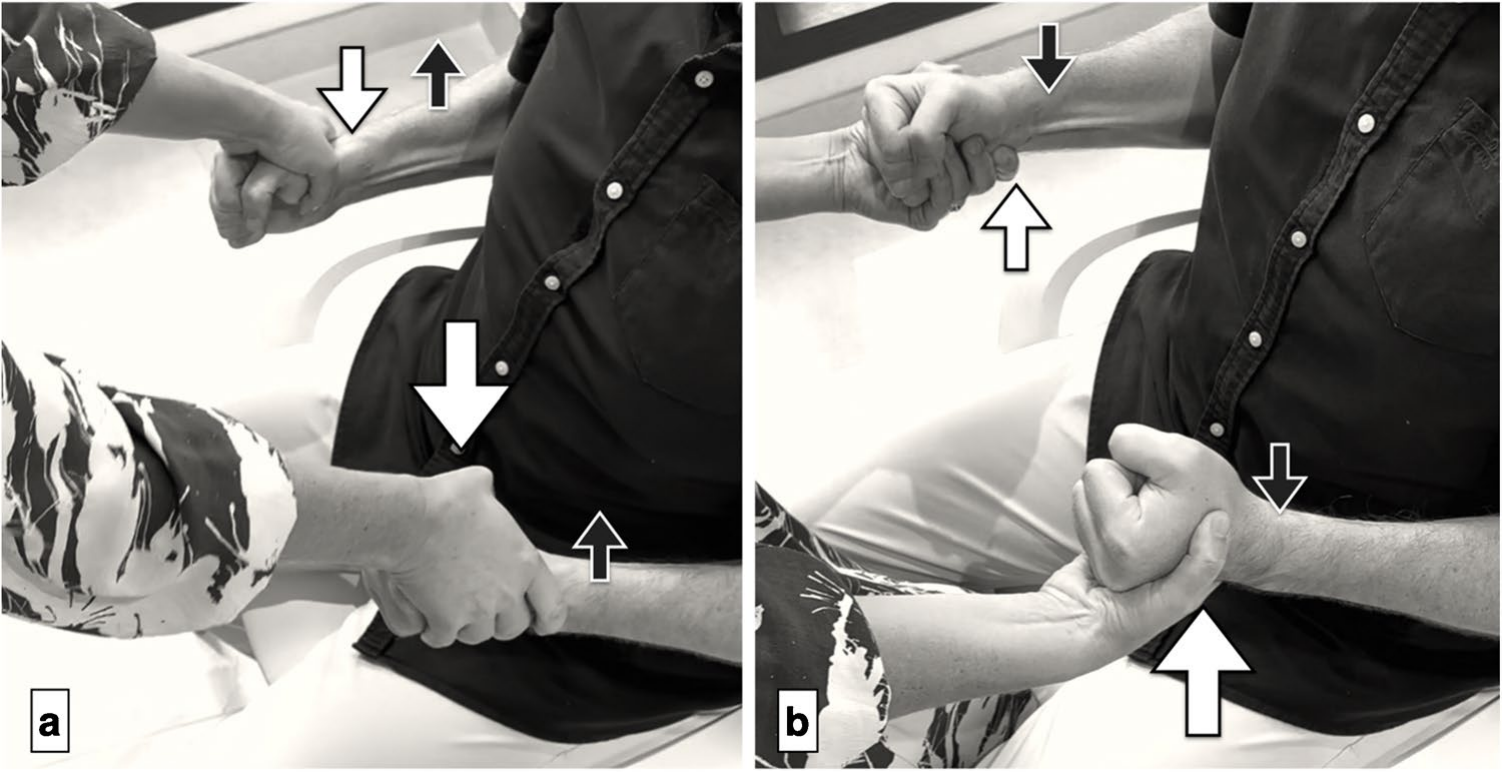
Muscle evaluation in elbow positions. a) For elbow flexion (biceps), the patient holds their elbows at 90 degrees with arms close to the body and actively flexes (black arrows) while the examiner applies downward resistance (white arrows). b) For elbow extension (triceps), with elbows bent at 90 degrees and arms adducted, the patient extends their elbows downward (black arrows) as the examiner exerts upward pressure (white arrows). *Reprinted with permission from Aspetar Journal*

**Fig. 3:**
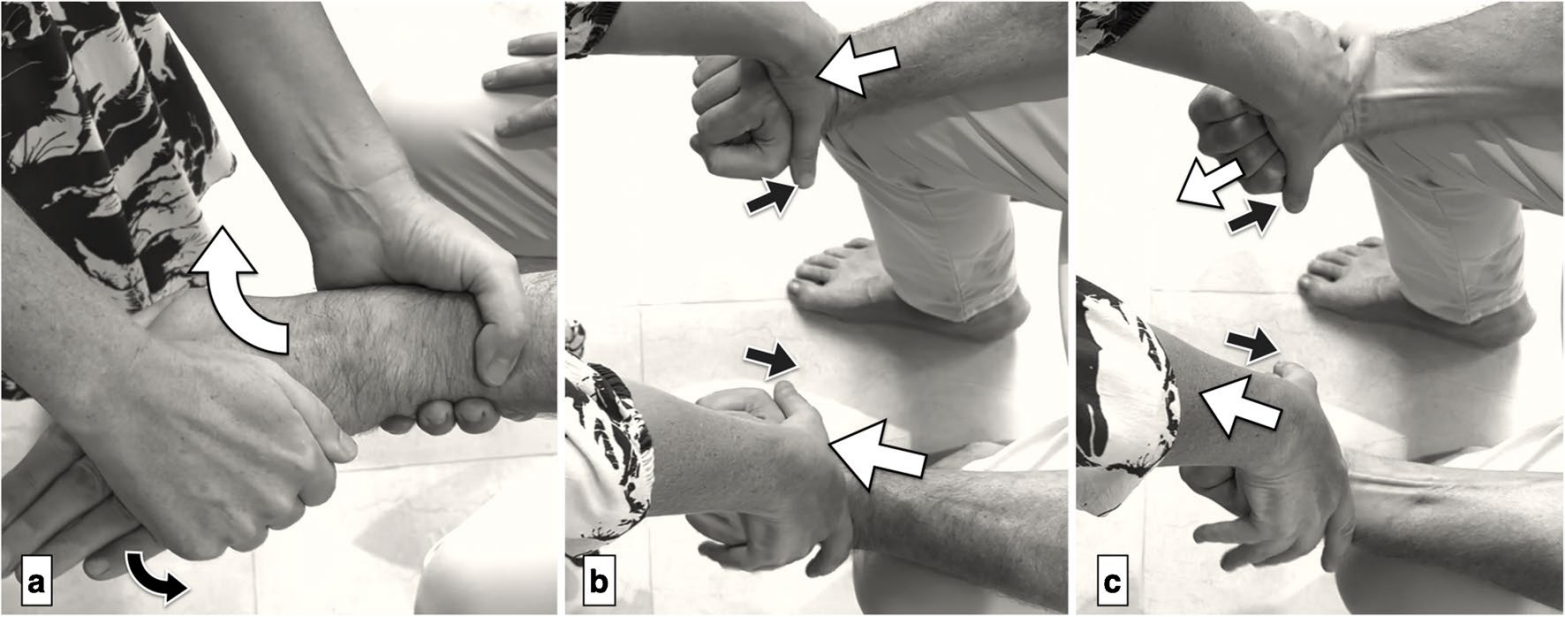
Muscle evaluation in wrist positions. a) Wrist ulnar deviation (ECU) is tested with the patient’s arm fully extended, wrist maximally deviated toward the ulnar side (black arrow), and the examiner stabilizing the forearm while pushing against the ulnar border of the hand (white arrows). b) Wrist extension (ECRB) is assessed by positioning the forearms pronated on the legs. The patient fully extends their wrist (black arrows) as the examiner applies force against the radial side of the hand (white arrows). c) For wrist flexion (FCR), the patient places their supinated forearms on their legs, flexes their wrist fully (black arrows), and the examiner counters by pushing against the radial side of the hand (white arrows). *Reprinted with permission from Aspetar Journal*

**Fig. 4:**
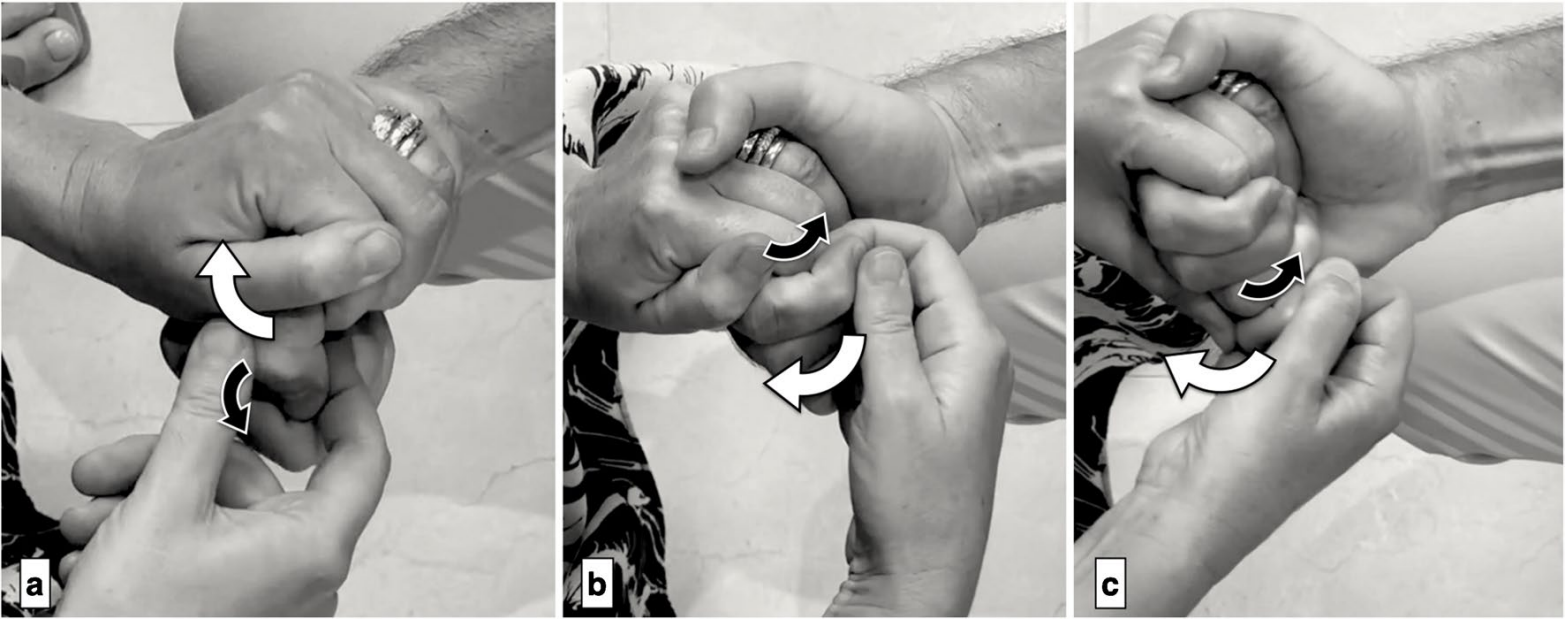
Muscle evaluation of hand extrinsics. a) Thumb flexion (FPL) is tested by stabilizing the thumb’s proximal phalanx while the patient flexes the interphalangeal (IP) joint (black arrows) and the examiner resists by applying force upward on the distal phalanx (white arrows). b + c) Flexion of the index and little fingers (FDP2, FDP5) is tested by isolating the distal interphalangeal (DIP) joints of these fingers with the wrist slightly flexed. The patient flexes maximally (black arrows), and the examiner resists with extension pressure (white arrows). *Reprinted with permission from Aspetar Journal*

**Fig. 5:**
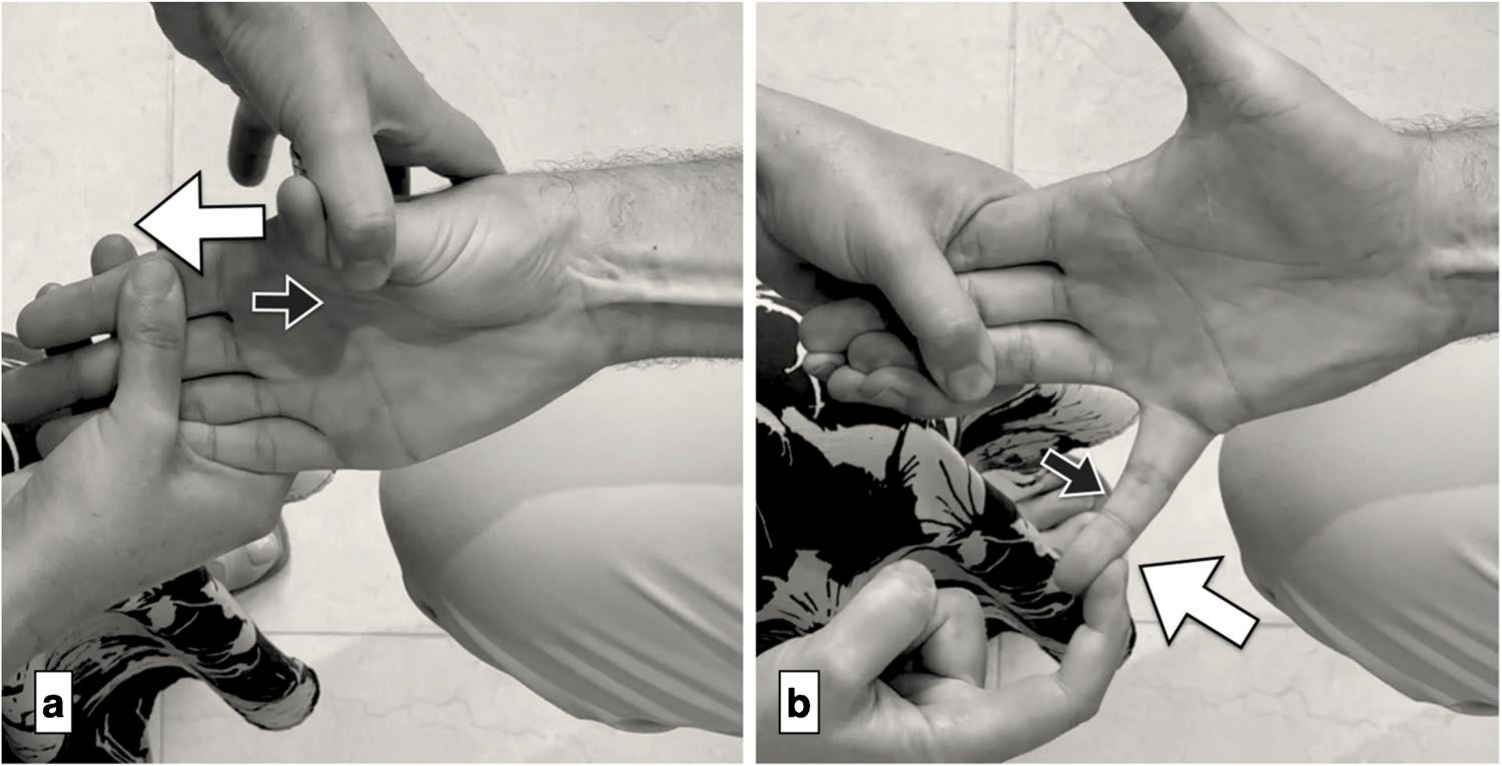
Muscle evaluation of hand intrinsics. a) Thumb abduction (APB) is assessed with the forearm in supination and the hand flat. The patient lifts the thumb upward (black arrows) while the examiner applies downward resistance (white arrows). b) Thumb abduction (ADM) is tested in the same forearm position, with the patient abducting the thumb maximally (black arrows) as the examiner applies resistance on the outer side of the little fingertip (white arrows). *Reprinted with permission from Aspetar Journal*

A standardized algorithm for upper extremity muscle testing has shown strong construct validity [7], good intra-rater reliability [8], and high sensitivity (88–92%) for identifying the level of nerve compression [9]. Testing involves assessing distinct patterns of muscle weakness distal to the site of nerve entrapment, with the following positions evaluated:

- **Shoulder**: Adduction (pectorals), abduction (posterior deltoid), and external rotation (infraspinatus).(Fig. 1)
- **Elbow**: Flexion (biceps) and extension (triceps). (Fig. 2)
- **Wrist**: Ulnar deviation (extensor carpi ulnaris, ECU), extension (extensor carpi radialis brevis, ECRB), and flexion (flexor carpi radialis, FCR). (Fig. 3)
- **Hand**: Extrinsic muscles (e.g., flexor pollicis longus (FPL), flexor digitorum profundus to the index (FDP2) and little finger (FDP5) (Fig. 4) and intrinsic muscles (e.g., abductor pollicis brevis (APB), abductor digiti minimi (ADM)). (Fig. 5)

General aspects of muscle testing include starting proximally and working your way distally in muscle testing; performing bilateral comparisons of strength; being consistent in testing; and applying adequate force. In patients with low-grade nerve compressions or in strong, muscular patients, it may also be necessary to do repeated testing of strength to assess for *fatigue*, commonly seen in dynamic nerve compression syndromes.

On a more specific note, when assessing the motor strength of the finger flexors, it is crucial to position the wrist in slight flexion while testing. This helps prevent false-positive strength readings in the FPL and FDPs, which can occur due to the tenodesis effect observed when the wrist is in neutral or slight extension.

The muscle testing can furthermore be added to additional muscles, depending on the intial findings from the screening above. As an example, weakness in wrist extension (ECRB) will often necessitate further testing of radial nerve innervated muscles, such as the thumb and index finger extension, to determine where the compression point is located (distal upper arm or forearm), see Table 3*.*

The muscle testing algorithm as it is used on a daily basis in clinical examination of any patient presenting with upper extremity pain, weakness and/or numbness is shown in video 1. An extended video describes the potential findings in each position, video 2*.*

## Strengths and limitations of MMT

The muscle testing algorithm has several strengths, including proven validity, high reliability, and sensitivity, as well as optimal accessibility, requiring only the clinician and patient. However, limitations exist, such as challenges in examining uncooperative patients, patients suffering from neurological disorders, or individuals with bilateral complaints. Additionally, subjectivity in testing, though reduced with experience, can also affect the results and interpretation of findings.

## The sensory-collapse test (SCT)

First described by Cheng, Beck, and Mackinnon in 2008 [10], as the scratch-collapse test, SCT is a valuable tool for verifying nerve entrapment levels. Its diagnostic accuracy in carpal and cubital tunnel syndromes is 82% and 89% [10], respectively, surpassing the commonly used Tinel's and Phalen’s flexion tests. In a systematic review of the sensitivity and specificity for diagnosis of nerve compressions syndromes, the general sensitivity is relatively low (38%), whereas the specificity is very high (94%) [11]. The conclusion of this is that SCT is a useful confirmatory tool that is most effective when used alongside other diagnostic methods, such as MMT, but is not recommended to be used as a stand-alone tool for nerve compression diagnosis [12].

The correlation between SCT and EDX was recently investigated in patients with carpal tunnel syndrome (CTS), where a positive SCT was correlated with positive EDX findings, in particular in cases if decreased median nerve conduction velocity and amplitudes, further supporting it’s use in diagnosis of nerve compressions [13].

The SCT has been proposed to be renamed the “sensory” collapse test instead of the “scratch” collapse test, as any sensory irritant can be used to provoke areas of allodynia. The physiology underlying the SCT is characterized by distinct reflex pathways that purposefully facilitate a protective response to external noxious stimuli [14]. In a pilot study investigating the utility of an “SCT-EMG,” a temporary loss of muscle tone, as measured by EMG, was seen following cutaneous stimulation of a compressed nerve [15]. Notably, this effect was absent after successful surgical decompression.

The SCT is performed by having the patient resist applied force before and after the examiner scratches/touches the skin over the suspected nerve compression site. A focal nerve compression results in a temporary loss of muscle strength and is called a “positive SCT” (video 3). Cold-spray (ethyl chloride) may also be used to confirm the site of compression by temporarily resolving the allodynia, resulting in a negative SCT [16].

## Testing for specific nerves of the upper extremity

As mentioned above, the superficial fascicles of the nerve at any compression point will usually generate motor or sensory deficits associated with a specific nerve compression syndrome. The axonal organization of the nerve at that point will, therefore, explain the patterns of weaknesses found in muscle testing [17].

## Clinical triad – median nerve compressions (Table 1)

### Anatomy

The median nerve originates from the cervical roots C6 to T1. Within the brachial plexus, it is formed by the union of the medial branch of the lateral cord and the lateral branch of the medial cord, creating the "median nerve fork." This junction occurs in the axilla or proximal portion of the upper arm. The nerve then travels along the medial aspect of the upper arm (Table 1).

**Table 1:** Median Nerve Compression Syndromes and the Clinical Triad

MEDIAN NECRVE TRIADS	Motor Weakness	SCT	Pain (compression site)
Lacertus Syndrome	FCR FPL FDP to index	Over the lacertus fibrosus	At the proximal edge of the lacertus fibrosus
Superficialis Syndrome	FDS to middle ring fingers	5–7 cm distal of the elbow crease, over the superficialis arcade	At the superficialis arcade
Anterior Interosseous Syndrome	FPL FDP to index PQ	Mid-forearm level, 2–3 cm distal of the superficialis arcade	Volar mid-forearm
Carpal Tunnel Syndrome	APB	At the level of the carpal tunnel	Wrist flexion crease (pos Tinels test)

*Abbreviatons: FCR* flexor carpi radialis, *FPL* flexor pollicis longus*, FDP* flexor digitorum profundus, *PQ* pronator quadratus, *APB* abductor pollicis longus

At the antecubital fossa, the median nerve passes through the lacertus tunnel [4], which is bounded anteriorly by the lacertus fibrosus and posteriorly by the trochlea humeri—an area prone to potential nerve compression. Beyond this point, the nerve may follow a variable path either adjacent to or through the pronator teres muscle. It then continues into the superficialis arcade, where the anterior interosseous nerve (AIN) branches off, supplying the flexor pollicis longus (FPL), the radial portion of the flexor digitorum profundus (FDP II-III), the pronator quadratus (PQ), and the volar wrist capsule [18].

The main median nerve extends through the distal forearm and, approximately 5 cm proximal to the wrist, gives rise to the palmar branch, which innervates the thenar eminence. It then continues its course through the carpal tunnel.

Compression of the medial cord in the infraclavicular space, for instance following an anterior shoulder dislocation, may result in weakness commonly associated with the median nerve. This site, however, is uncommon as a cause of isolated median nerve compression per se.

### Lacertus syndrome

Far more common are the compressions of the median nerve (MN) in the proximal forearm, collectively called proximal median nerve entrapments (PMNE). Recent publications have shown that one of the primary compression points is at the level of the lacertus fibrosus (LF), in what is known as the *lacertus syndrome* [19, 20].

- *Muscle testing:* Dynamic or static compression of the nerve at this level results in weakness in the FCR, FPL, FDP2.‬‬‬‬‬‬‬
- *Sensory testing*: Testing is directed to the area from the ulnovolar antecubital fossa to the LF‬‬‬‬‬‬‬
- *Pain:* Pain will be present at the level of the LF.‬‬‬‬‬‬‬

### Superficialis syndrome

Five to seven centimeters distal of the antecubital fossa, the median nerve can be compressed at the superficialis arcade, so-called *superficialis syndrome*.

- *Muscle testing:* Weakness will be seen in the flexor digitorum superficialis to the middle- and ring fingers (FDS III-IV).‬‬‬‬‬‬‬
- *SCT:* Testing is directed to the area of the arcade, approximately four fingerbreadths distal of the elbow crease.‬‬‬‬‬‬‬
- *Pain:* Positive at the level of the arcade.‬‬‬‬‬‬‬

### Anterior interosseous nerve syndrome

Rarely seen, the anterior interosseous nerve syndrome entails a compression of the AIN after the bifurcation from the median nerve [21].

- *Muscle testing:* As with the lacertus syndrome, weakness will be seen in the FPL, FDP II but not in the FCR. Pronator quadratus weakness may be present, but is often difficult to isolate and test due to strength in the pronator teres.‬‬‬‬‬‬‬
- *SCT:* Testing is directed to the mid-forearm level‬‬‬‬‬‬‬
- *Pain:* Positive about 2–3 cm distal of the superficialis arcade, mid-forearm‬‬‬‬‬‬‬

### Carpal tunnel syndrome (CTS)

CTS is by far the most commonly diagnosed and described nerve compression. As such, there are a myriad of clinical examination tests for this diagnosis, in addition to nerve conduction tests [5]. For this article, only the findings in line with the clinical triad are described.

- *Muscle testing:* Thenar atrophy may be seen in advanced CTS. As most patients present with earlier stages of nerve compression, a subtle weakness in the APB is most commonly found.‬‬‬‬‬‬‬
- *SCT*: A positive test will be elicited at the proximal edge of the carpal tunnel.‬‬‬‬‬‬‬
- *Pain:* CTS is only sometimes associated with pain on compression. Rather, the triad is supported by performing Tinel’s test, where percussion of the median nerve at the carpal tunnel will result in paresthesias into the median innervated area of the hand.‬‬‬‬‬‬‬

## Clinical triad – ulnar nerve compressions (Table 2)

### Anatomy

The ulnar nerve originates from the C8-T1 nerve roots and forms the medial cord of the brachial plexus. The medial cord divides into four branches: the medial brachial cutaneous, the medial antebrachial cutaneous, a branch to the median nerve, and a branch continuing as the ulnar nerve. The ulnar nerve travels along the medial arm and passes posterior to the medial epicondyle through the “cubital tunnel.” The dorsal cutaneous branch of the ulnar nerve separates from the main trunk approximately 5 cm proximal to the ulnar styloid [22]. Distally, the ulnar nerve crosses the wrist alongside the artery and passes through Guyon’s canal. Within Guyon’s canal, the nerve divides into the deep motor and superficial sensory branches. The deep motor branch travels beneath the pisohamate arch [23], (Table 2).

**Table 2:** Ulnar Nerve Compression Syndromes and the Clinical Triad

ULNAR NERVE TRIADS	Motor Weakness	SCT	Pain (compression site)
C8-radiculopathy	FCU FDP little finger Intrinsic hand muscles	Level of the C8, back of the neck	No specific pain point on compression; generalized pain on neck movement; radicular pain
Thoracic outlet syndrome (TOS)	FDP to index FPL Intrinsic hand muscles	Scalene interval, side of the neck	Pain at the lower scalene interval on the side of the neck
Cubital tunnel syndrome	FCU FDP small finger Intrinsic hand muscles	Staged SCT from proximal to distal of the cubital tunnel to confirm level of compression	Pain test based on SCT finding Common: distal of the cubital tunnel
Ulnar tunnel (Guyon’s canal)	Ulnar intrinsic hand muscles, especially ADM FDI	Ulnovolar wrist crease and/or hook of hamate	Proximal of Guyon’s canal and/ or hook of hamate

*Abbreviatons: C8* cervical level 8, *FCU* flexor carpi ulnaris, *FPL* flexor pollicis longus, *FDP* flexor digitorum profundus, *ADM* abductor digiti minimi, *FDI* first dorsal interosseous

Ulnar-sided numbness in the hand can be a result of compression anywhere from the C8 level, through the thoracic outlet to the hand. In addition to the clinical triad, a careful assessment of the distribution of numbness – ulnar hand, ulnar forearm, ulnar upper arm, shoulder – is essential to stage the level of nerve affliction in these patients [18].

### Cubital tunnel syndrome

The ulnar nerve can be compressed at various locations around the elbow, with the most frequent site being at Osborne’s ligament [24]. Other potential areas of compression include the arcade of Struthers [25], the medial intermuscular septum located proximal to the cubital tunnel, and the flexor carpi ulnaris (FCU) arcade.

- *Muscle testing:* Weakness is consistently found in FDP V. At times it is also possible to isolate weakness in the FCU.‬‬‬‬‬‬‬
- *SCT:* Most common site of entrapment is in the distal cubital tunnel, at the level of Osbornes ligament and the FCU arcade. SCT is an excellent tool to help stage the level of compression at the cubital tunnel and should be performed at all potential sites of compression [24]‬.‬‬‬‬‬‬
- *Pain:* Pain site is dependent on compression site, most commonly pain will be noted at the FCU arcade and Osbornes ligament.‬‬‬‬‬‬‬

### Guyon’s canal – ulnar tunnel syndrome

The symptoms associated with compression in Guyon's canal vary based on the specific location [26]. Compression in *zone I* (Guyon’s canal proper) may cause both motor and sensory deficits, while *zone II* (deep branch of the ulnar nerve) affects motor function only, and *zone III* (ulnar of zone II) leads to sensory symptoms exclusively.

- *Muscle testing:* Important to test ADM and IOD1. Both muscles will be weak in zone I whereas only IOD1 in zone III compression.‬‬‬‬‬‬‬
- *SCT:* Staged SCT based on muscle testing, will be positive in either zone I, II or III.‬‬‬‬‬‬‬
- *Pain:* Also staged and should be correlated to motor and SCT findings for zones I, II, III.‬‬‬‬‬‬‬

## Clinical triad – radial nerve compressions (Table 3)

### Anatomy

The radial nerve arises from the posterior cord (cervical roots C5-T1) of the brachial plexus. It travels along the posterior aspect of the shoulder and upper arm, then passes into the anterior compartment of the distal forearm through the lateral intermuscular septum (LIS). Continuing its path, it runs deep beneath the brachioradialis (BR) before dividing into the posterior interosseous nerve (PIN) and the sensory branch of the radial nerve (SBRN). Compression of the radial nerve can occur at multiple points, including two locations in the upper arm, two in the proximal forearm, and one in the distal forearm/wrist [27], (Table 3).

**Table 3:** Radial Nerve Compression Syndromes and the Clinical Triad

RADIAL NERVE TRIADS	Motor Weakness	SCT	Pain (compression site)
Triangular Interval Syndrome (TIS)	Triceps ECRB EIP EPL	Posterior upper arm, just distal of the axilla	Just distal of the axilla and proximal of the radial (spiral) groove
Lateral Intermuscular Syndrome (LIS)	ECRB EIP EPL	Lateral aspect of the distal upper arm	9–10 cm proximal of the lateral epicondyle
Posterior Interosseous Nerve (Radial Tunnel) Syndrome	ECU (EDM)	Posterolateral aspect of proximal the forearm	Three fingerbreadths distal of the lateral epicondyle, over the arcade of Frohse
Brachioradialis Syndrome (BRS) -“High” Wartenberg	ECRB	Radiovolar aspect of the proximal forearm, just distal of the radiovolar elbow crease	Volar proximal edge of the brachioradialis
”Low” Wartenberg Syndrome	No motor weakness	9–10 cm proximal of the radial styloid, along the radial border of the forearm	Painful positive Tinel’s test where the nerve exists between ECRL-BR

*Abbreviatons: ECRB* extensor carpi radialis brevis, *EIP* extensor indicis proprii, *EPL* extensor pollicis longus, *ECU* extensor carpi ulnaris, *EDM* extensor digit minimi, *BR* brachioradialis

### Triangular interval syndrome (TIS)

TIS is compression of the radial nerve in the triangular interval on the posterior aspect of the shoulder, as the nerve passes between the long and lateral heads of the triceps.

- *Muscle testing:* Weakness in all radial innervated muscles, most easily identified as weakness in triceps and wrist extension.‬‬‬‬‬‬‬
- *SCT:* Tested in the posterior upper arm, just distal of the axilla.‬‬‬‬‬‬‬
- *Pain:* Pain is found just distal of the axilla and proximal of the radial (spiral) groove.‬‬‬‬‬‬‬

### Lateral intermuscular syndrome (LIS)

After passing through the spiral groove of the humerus, the radial nerve pierces the lateral intermuscular septum approximately 9–10 cm proximal of the lateral epicondyle, to exit between the brachialis and BR.

- *Muscle testing:* Weakness in wrist extension, index finger (extensor indicis proprii, EIP) and thumb (extensor pollicis longus, EPL).‬‬‬‬‬‬‬
- *SCT:* Tested in the lateral aspect of the distal upper arm‬‬‬‬‬‬‬
- *Pain:* At the level of the nerve piercing the lateral intermuscular septum, 9–10 cm proximal of the lateral epicondyle.‬‬‬‬‬‬‬

### Radial tunnel syndrome (RTS)

The posterior interosseous nerve (PIN) is prone to compression under the proximal supinator edge, aka the arcade of Frohse. RTS is frequently misdiagnosed as lateral epicondylitis and commonly presents as dull, aching forearm pain in the mobile extensor wad [28].

- *Muscle testing:* The superficial fascicles in the PIN are to the extensor carpi ulnaris (ECU), which is tested by having the patient extend the arm fully and ulnarly deviate the hand maximally, while the examiner tries to forcefully radially deviate the wrist [29]‬.‬‬‬‬‬‬
- *SCT:* The SCT is directed to the posterolateral aspect of the forearm, over the arcade of Frohse.‬‬‬‬‬‬‬
- *Pain:* Deep pressure is applied approximately three fingerbreadths distally and obliquely from the lateral epicondyle, over the arcade of Frohse.‬‬‬‬‬‬‬

### Brachioradialis – “high” Wartenberg syndrome

The “high” Wartenberg syndrome has recently been described as a compression of the SBRN in the proximal forearm, under the edge of the BR [30]. While the sensory branch of the radial nerve (SBRN) is typically considered a purely sensory nerve, recent anatomical research suggests that it frequently provide motor innervation to the ECRB [31].

- *Muscle testing:* Weakness, if present, is only found in wrist extension (ECRB).‬‬‬‬‬‬‬
- *SCT:* Testing is directed to the radiovolar aspect of the proximal forearm, just distal of the radiovolar elbow crease.‬‬‬‬‬‬‬
- *Pain:* Deep pressure over the volar proximal edge of the brachioradialis will elicit pain.‬‬‬‬‬‬‬

### “Low” Wartenberg syndrome

Representing a pure sensory nerve involvement, the “low” Wartenberg syndrome involves compression of the SBRN as the nerve exists in the distal forearm, approximately 9–10 cm proximal of the radial styloid, between the extensor carpi radialis longus (ECRL) and BR [32].

- *Muscle testing:* No motor weakness is found‬‬‬‬‬‬‬
- *SCT:* Positive approximately 9–10 cm proximal of the radial styloid, along the radial border of the forearm.‬‬‬‬‬‬‬
- *Pain:* Painful positive Tinel’s test where the nerve exists between ECRL-BR.‬‬‬‬‬‬‬

## Lower extremity clinical triads

The clinical triad is also applicable to the lower extremities, although identifying M4 weaknesses can be challenging due to the size and strength of lower limb muscles. Distal muscle testing, particularly in the ankle and foot, can help identify nerve compressions around the knee or ankle [33].

The most commonly found lower extremity nerve compressions involve the peroneal nerve, and will be used as an example of clinical triad for lower extremity.

## Clinical triad – peroneal nerve compressions (Table 4)

### Anatomy

The peroneal nerve originates from the sciatic nerve (spinal roots L4-S2), just proximal of the popliteal fossa. After traveling posterior of the distal tendon of the biceps femoris, it curves around the fibular neck, to enter the peroneal tunnel in the lateral compartment of the lower limb. Just distal of the peroneal tunnel, the nerve divides into the deep and superficial peroneal nerves. The deep peronal nerve contributes motor branches to the tibialias anterior (TA) and extensor hallucis longus (EHL), whereas the superficial peroneal nerve gives motor branches to the peronal muscles and sensation to the dorsum of the foot (Table 4).

**Table 4:** Peroneal Nerve Compression Syndromes and the Clinical Triad

PERONEAL NERVE TRIADS	Motor Weakness	SCT	Pain (compression site)
Common peroneal nerve syndrome (CPN)	TA EHL Peroneus longus and brevis	Proximal lateral leg, at the fibular head	No specific pain point on compression; generalized pain on neck movement; radicular pain
Superficial peroneal nerve syndrome (SPN)	No motor weakness	Lateral distal lower leg, approximately 9–10 cm proximal of the lateral malleolus	Painful positive Tinel’s test, 10 cm proximal and slightly anterior of the lateral malleolus

*Abbreviatons*: *TA* tibialis anterior, *EHL* extensor hallucis longus

### Common peroneal nerve (CPN) compression syndrome

CPN is a frequently underdiagnosed compression syndrome, but an important cause of trip and fall injuries in the elderly [34].

- *Muscle testing:* Weakness is found in ankle extension (TA), big toe extension (EHL) and ankle eversion (peroneal muscles).‬‬‬‬‬‬‬
- *SCT:* Testing is directed to the proximal lateral leg, over the fibular head [35].‬‬‬‬‬‬‬
- *Pain:* Painful positive Tinel’s test where the nerve curves around the fibular head. It is easy to palpate the CPN at this level, and even superficial palpation is often uncomfortable.‬‬‬‬‬‬‬

### Superficial peroneal nerve (SPN) compression syndrome

SPN compression syndrome is often seen following ankle sprain injuries, resulting in a chronic ankle pain with numbness over the dorsal aspect of the foot [36]. The compression point is where the SPN exists under the deep transverse fascia to become superficial, approximately 9 cm proximal of the lateral malleolus and 3 cm lateral of the anterior tibial crest [37].

- *Muscle testing:* No muscle weakness as the compression point is distal of the motor branches to the peroneal muscles.‬‬‬‬‬‬‬
- *SCT:* Testing directed to the lateral distal lower leg, approximately 9–10 cm proximal of the lateral malleolus.‬‬‬‬‬‬‬
- *Pain:* Painful positive Tinel’s test where the nerve exists under the deep transverse fascia.‬‬‬‬‬‬‬

## Double-crush and multiple-crush syndromes

The examination of any patient with pain, numbness, and/or weakness also implies keeping an open mind to the possibility of more than one nerve compression existing at any one time.

The concept of double-crush syndrome entails the finding of more than one nerve compression along the same nerve [38], where one of the most common double-crush sites includes the concomitant carpal tunnel- and lacertus syndromes [39].

Multiple-crush syndromes are frequently seen in patients with underlying metabolic disorders, such as diabetes mellitus or even following trauma [40]. A recent study highlighted this concept by investigating the presence of carpal tunnel syndrome (CTS) and fall injuries, indicative of common peroneal nerve compression (CPN), suggesting that any patient with CTS diagnosis should also be screened for possible CPN as an early diagnosis can offer an easily treatable way to avoid future fall injuries [41].

A clinician should thus be consistent in the use of the clinical triad, examining from proximal to distal in all patients with complaints of numbness, weakness or pain, as compressions may exist on multiple levels, indeed in both upper- and lower extremities.

## Conclusion

Nerve compression syndromes can significantly impact daily life, causing pain, muscle weakness, and loss of function in the affected limb. A structured approach to the examination of patients with potential nerve entrapments is essential for effective diagnosis and management. The clinical triad—manual muscle testing, the sensory-collapse test, and pain assessment—provides a structured framework for identifying nerve compressions, even when electrodiagnostic and imaging studies are normal. Although the clinical triad has been most reported in relation to upper extremity nerve compressions, it is also possible to implement in cases of lower extremity nerve disorders. A complete testing of muscle function, SCT, and pain takes a few minutes in the clinic, making it an easy screening tool for any clinician dealing with musculoskeletal and peripheral nerve pain disorders.

## Supplementary Information

Below is the link to the electronic supplementary material.

Supplementary file1. Video 1 Full-length video of the manual muscle testing technique for screening of upper extremity nerve compressions, including explanations of possible findings. (MP4 150090 KB)

Supplementary file2. Video 2 Short video showing the muscle testing technique as used in daily practice, screening from shoulder to hand. (MP4 27273 KB)

Supplementary file3. Video 3 The sensory-collapse test (SCT) as seen in a patient with right-sided lacertus syndrome and left-sided cubital tunnel syndrome. (MP4 29711 KB)

## Data Availability

No datasets were generated or analysed during the current study.
